# Effects of maternal dietary energy restriction on laying performance, embryonic development, and lipid metabolism in broilers

**DOI:** 10.5713/ab.21.0301

**Published:** 2021-11-01

**Authors:** Hao Sun, Zhihui Chen, Chengzhan Ma, Lina Lian, Zeyu Zhao, Shupeng Niu, Liangmei Xu, Jinhua Sun

**Affiliations:** 1 College of Animal Science and Technology, Northeast Agricultural University, Harbin, Heilongjiang 150000, China

**Keywords:** Broiler Breeder, Embryo, Energy Restriction, Lipid Deposition, Lipoprotein Lipase

## Abstract

**Objective:**

The objective of this study was to investigate the effects of different degrees of maternal dietary energy restriction on lipid deposition in embryonic tissues during the medium laying period (37 to 39 weeks) in Arbor Acres (AA) broiler breeders.

**Methods:**

A single factor design was adopted, and 400 AA broiler breeders (20 weeks of age) with a similar weight were randomly allocated into four groups. The birds in the control group were fed a corn-soybean meal based diet, and those in trial groups were fed diets with 80%, 70%, and 50% energy levels of the basal diet. Incubated eggs from the medium laying period were collected. Samples of developing embryos at various stages were prepared for composition analysis.

**Results:**

The embryo weight in the 80% energy group was higher than those of the other groups on embryonic day (E) 13, but at 21 E, they were significantly decreased with decreasing energy intake of the broiler breeders (p<0.05). Additionally, the levels of crude fat in tissues in the restriction groups were significantly decreased (p<0.05). The long axis and area of adipocytes in breast muscle, thigh muscle and the liver were significantly decreased (p<0.05) at 21 E in the 80%, 70%, and 50% energy groups.

**Conclusion:**

The effects of the 80% maternal dietary energy restriction energy affects egg production performance, egg quality, and nutrient deposition in egg weights, which then directly impacts on the developmental process of embryos, especially on fat utilization and deposition.

## INTRODUCTION

Energy and protein restriction have been used for layers to avoid excessive intake which can lead to bird fatness and increase feeding costs [1]. Numerous investigations have focused on methods to increase egg weight through diet manipulation. So, it is necessary to have a better understanding on how to optimize the use of dietary energy to get optimal performance and profits of laying hens. In addition, Blamberg et al [2] have found that metabolizable energy has a great effect on egg weight. Furthermore, many animal studies demonstrated that excess intake of energy affects the growth rate and could negatively affect the carcass characteristics [3]. Therefore, it is important to control the energy intake. Additionally, maternal nutrition during gestation is closely associated with offspring growth and development in animals, which likely affects mRNA transcription, hormone secretion, antibodies, permeability of the placenta, breast milk composition, maternal parenting behavior and so on [4]. Gardner et al [5] reported that increasing dietary protein of the hens from 12% to 18% significantly increased whole egg, yolk, albumen, and shell weights. Shafer et al [6] found that elevated dietary methionine could increase egg size, component mass, and solids content of albumen and yolk. The structural and functional changes during offspring development submitted to nutritional restriction may be explained by phenotypic accommodation, which is an adaptive adjustment [7,8]. Thus, it is necessary to maintain the maternal energy balance during embryo development.

Unlike mammals, embryogenesis in birds must rely on the nutrients provided by the egg, which is mainly influenced by maternal conditions [9]. It has been demonstrated that hatching chicks are affected by the maternal diet and metabolism of hens [10,11]. Dietary energy is one of the most important components in poultry rations [12]. Additionally, high lipid intake is harmful to animal health [13], and Enting et al [14] showed that low-energy broiler breeder diets during the rearing phase not only increases egg production and affects the embryonic development, performance and mortality of their offspring but they also improve offspring growth rates and reduce mortality. Our previous studies have reported that maternal dietary energy restriction could significantly increase serum total cholesterol (CHO) in embryos during the middle laying period in broiler breeders [15]. These previous studies demonstrated that dietary energy restriction of the hens is helpful to promote differentiation of pre-adipocytes of the embryos, but we still do not know whether an 80% maternal dietary energy restriction is the most appropriate diet or not. Then, it is essential to deeply and systematically analyze the effect of different levels of maternal dietary energy restriction. In this study, we continue to investigate the effects of maternal dietary energy restriction with 80%, 70%, and 50% energy levels of the basal diet during the medium laying period in broiler breeders. This research comprehensively evaluates the effect of maternal dietary energy restriction on lipid deposition. Meanwhile, it also provides a scientific theoretical basis and technical support to further research of maternal effects.

## MATERIALS AND METHODS

### Animal care

This study was approved by the Animal Care and Use Committee of Northeast Agricultural University. Animals used in this experiment were cared for under the guidelines stated in the Guide for the Care and Use of Agricultural Animals in Agricultural Research and Teaching of Heilongjiang Province (Approval Number: NEAU-2011-9).

### Animals and experimental design

Four hundred of 20-week-old Arbor Acres (AA) breeder hens, with no significant difference in initial weight, were randomly assigned into 4 treatments (5 replicates of 20 birds per treatment). In the control group, hens were fed a corn-soybean meal based diet. The trial lasted for 25 weeks, and incubated eggs from the medium laying period (37 to 39 weeks) were collected and analyzed to investigate the effects of maternal dietary energy restriction during the medium laying period. The basal diet was formulated in accordance with NY/T 33–2004 (Chicken Feeding Standard, Agricultural industry standard of the P. R. China). Those in the trial groups were fed the diets with 80%, 70%, and 50% energy levels of the basal diet. The nutrient levels of each diet were the same, except for metabolizable energy (Table 1).

### Feeding management

Hens were kept in three-layer complete ladder cages at 2 birds per cage (36 cm×25 cm×39 cm) equipped with water nipples, and ten cages of same treatment were placed together. During the test period, all hens were restrictively fed at 08:00 hours each day and supplied with water *ad libitum* (Table 2). A 16-h photoperiod and 8 h darkness was maintained throughout the experiment. Average temperature in the chicken house was 20°C±0.5°C, and sterilization and epidemic prevention in the room were conducted regularly. During the entire laying period, artificial insemination was conducted in broiler breeders every 5 days. Semen was taken from one large pool from multiple roosters so that all hens were inseminated with the same semen mixture. Eggs were marked and stored in a dark room at 10°C until incubation. At the end of collection period, eggs were segregated into groups and placed into an FT-ZF10 automatic incubator with altering incubating temperatures and turning the eggs every two hours. The incubating temperature was maintained at 38.4°C from day 1 to 6 days, 38.1°C from 7 to 12 days, 37.2°C from 13 to 18 days, and 36.9°C for the next three days during the incubation period. Humidity was controlled in range from 60% to 75% by adding water.

### Data and sample collection

During the test before the hatching period, egg production, egg weight and feed consumption were recorded daily in replicate for calculation of laying rate, average egg weight, average daily feed intake and feed conversion ratio. The weights of egg content and embryo were recorded on day 13 E, 15 E, 17 E and 19 E. Per treatment, twenty eggs with a properly developed embryo were sampled to determine the embryonic lipid deposition in tissue. Each egg was weighed and then broken. Egg yolk and egg white separators were used to separate the egg yolk and egg white, and then freeze-dried with a lyophilizer (LYOQUEST-85; Azbil Telstar Technologies, S.L.U. Spain) and Kjeldahl (KT-2300; FOSS, Hoganas, Sweden). The crude protein content of air-dried egg yolk and egg white were determined, and the coefficient of calculation was 6.25. The content of crude fat in egg yolk and egg white was determined by ether extraction. The weights of 1-day-old offspring were recorded on day 21 E. Whole embryo, livers, breast muscle and thigh muscle were dissected and then preserved at −80°C at 13 E, 15 E, 17 E, 19 E, and 21 E. The eggs without any embryo inside were not sampled.

The contents of crude fat of the yolk were measured by the soxhlet extraction method. The total energy of the yolk was determined using a Parr 6300 bomb calorimeter (Parr Instrument Company, Moline, IL, USA). The total energy was reported in megajoules as the unit mass of a solid biofuel burned in oxygen in a calorimetric bomb under specified conditions. The cholesterol in the yolk was determined using the CHOD-PAP method (Richmond, 1973) and a total cholesterol assay kit (BHKT Clinical Reagent Co., Beijing, China). The kit contained a cholesterol assay reagent and standard cholesterol solution, which was used for the calibration curve. An ultraviolet-visible spectrophotometer UV-2401PC (Shimadzu co., Kyoto, Japan) was used to measure the sample absorbance.

Breast muscle, thigh muscle and liver on the left side of each bird were collected and stored in 2.5% dilute glutaral solution for electron microscopic section. Analytical samples were prepared by dropping diluted nanotube dispersion in water onto carbon-coated copper grids and allowing the water to evaporate. The different treatment samples were cut into 2-mm^2^ sections and fixed standard paraffin embedding to measure the diameter, area and density of adipocytes using HITACHI H-7650 transmission electron microscope (TEM). The total RNA was isolated from the organs using a reagent box (E.Z.N.A. Total RNA Kit; Omega Bio-tek, Inc., Doraville, GA, USA) according to the manufacturer’s recommended protocol. The concentration of RNA was estimated based on the absorbance at 260 nm, which was determined using a spectrophotometer. Then, the lipoprotein lipase (LPL) expression levels were determined through quantitative real-time polymerase chain reaction (PCR) with an ABI PRISM 7500 SDS thermal cycler apparatus (Applied ABI PRISM 7500 Real-Time PCR System). Primers were designed from published GenBank sequences and were synthesized by Sangon (Table 3). For analyses on an ABI PRISM 7500 SDS thermal cycler, PCR reactions were performed with 2.0 mL of first-strand cDNA and 0.4 mL of sense and anti-sense primers in a final volume of 20 mL. Samples were centrifuged briefly and run on the PCR machine using the default fast program (1 cycle at 95°C for 30 s, 40 cycles of 95°C for 5 s and 60°C for 34 s). All the PCR reactions were performed in triplicate. The relative gene expression levels were determined using the 2–ΔΔCt method [16].

### Statistical analysis

Statistical analyses were performed using Statistical Analysis System (SAS) version 9.4. One-way analysis of variance (ANOVA) was used for statistical analysis. Data are expressed as means±standard error of the mean. Statistical comparisons of different treatments were performed using one-way ANOVA. The test results of all analyses were considered significant when p<0.05.

## RESULTS

The results of the indexes on the egg performance of broiler breeders are shown in Table 4. The laying rates of the 70% and 50% energy group were significantly lower than that of the control group (p<0.05), but the 80% energy group showed no significant difference with the control group (p>0.05), the qualified egg rates of the 80%, 70%, 50% energy group were significantly higher than that of control group (p<0.05), egg weight and crack egg rates were significantly lower than that of control group (p<0.05) (Table 4).

The results of the indexes on the restriction on hatching performance are shown in Table 5. Compared with the control group, the fertility rate was significantly increased by 80% and 70% of restricted feeding group of the hens (p<0.05), but it was significantly reduced by the 50% restricted group (p<0.05). Compared with the control group, the incubation rate was significantly reduced by 50% energy group (p<0.05), but there was no significant effect on 80% or 70% energy group (p>0.05). In addition, the restricted energy of hens has no significant effect on the healthy rate of the population (p>0.05) (Table 5).

The results of the indexes on the egg component are shown in Table 6 and 7. Compared with the control group, the yolk of each restricted feeding group decreased significantly (p<0.05). The relative weight of egg whites significantly increased (p<0.05). There was no significant effect on the relative weight of the shell, yolk, fat of egg yolk and the dry matter of the egg white (p>0.05). Compared with the control group, the total cholesterol of egg yolk in the 70% energy group was significantly reduced (p<0.05), in addition, the total cholesterol and egg white protein in the 50% energy group were significantly reduced (p<0.05) (Tables 6, 7).

The results of the indexes on the development of embryo are shown in Table 8. The result of Embryo weight/incubation weight showed that the relative embryo weight increased with the aging of the embryo. And the relative embryo weight in the 80% energy group was significantly higher than that of other groups (p<0.05) on 13 E. On 21 E, the relative embryo weight was decreased significantly by all the restricted groups (p<0.05). On 13 E, the embryo weight and thigh muscle weight in the 80% energy group was significantly higher than other groups (p<0.05); the breast muscle weight in the 80% energy group was significantly increased compared to that of the control and 50% energy groups (p<0.05). On 17 E, the thigh muscle weight in the 80% energy group was significantly improved compared to the control group (p<0.05). The hatched chick with the yolk weight at 21 E was significantly decreased with the decreasing of dietary energy level (p<0.05). There was no significant difference on liver weight in the four groups on 13 E–21 E (p>0.05) (Table 8).

The lipid content of yolks during the hatching period is shown in Table 9. The total cholesterol levels of yolks on 19 E and 21 E were abruptly reduced compared to the previously tested days. The total cholesterol levels on 13 E and 17 E of every restriction group were higher than control group (p<0.05). Maternal dietary energy level did not affect the crude fat and gross energy level in embryonic yolks (p>0.05).

Tissue composition in the embryo (Table 10) demonstrated that the total cholesterol content in the breast muscle on 13 E and the thigh muscle on day 15 E in the 80% energy group was significantly higher than other groups (p<0.05). The total cholesterol in the thigh muscle for the 80% energy groups on 19 E was significantly decreased compared to other groups (p<0.05). The total cholesterol in the thigh muscle on 17 E was significantly decreased (p<0.05) in the energy restriction groups. The total cholesterol in the liver on 13 E and 15 E was also significantly decreased (p<0.05) in the energy restriction groups. The crude fat in breast muscle on 17 E and 19 E as well as in thigh muscle on 21 E and in the liver on 15 E were significantly decreased (p<0.05) in the 80%, 70%, and 50% energy groups compared with the control group (Tables 9, 10).

The results of the long axis, area, and quantity of adipocytes in breast muscle, thigh muscle and the liver are shown in Tables 11–13. The long axis in breast muscle were significantly decreased (p<0.05) at 13, 17, 19, and 21 E in the 80%, 70%, and 50% energy groups compared with the control group. The long axes in breast in the 70% and 50% energy groups were significantly decreased than control group (p< 0.05). The area of adipocytesin breast muscle was significantly decreased at 17, 19, and 21 E. Compared with the control group, the long axis and area of adipocytes in the thigh muscle were significantly decreased (p<0.05) on 21 E in the 80%, 70%, 50% energy groups. Long axis in the thigh muscle was significantly decreased (p<0.05) on 17 E in the 70%, 50% energy groups. Compared with control group, the long axis and area of adipocytes in the liver were significantly decreased (p<0.05) on 21 E in the 80%, 70%, 50% energy groups, and the area was significantly decreased on 17 E (p<0.05). There was no significant difference in the number of adipocytes in breast muscle, thigh muscle and the liver (p>0.05) (Tables 11, 12, 13).

**Figure 1:**
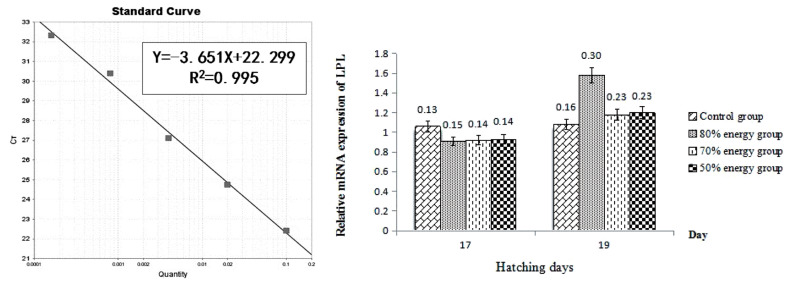
The amplification efficiency curve of lipoprotein lipase (*LPL*) gene by real time polymerase chain reaction (left); effects of maternal energy restriction on the expression of LPL mRNA in breast muscle of embryo at 17 E and 19 E (right).

The effects of the maternal dietary energy levels on the relative ratios of LPL expression are shown in Figure 1. The LPL expression in the 80% energy group on 19 E showed an increasing trend (p>0.05) (Figure 1).

## DISCUSSION

Feed intake and egg weight can significantly affect cost of production and profits. A few studies have suggested that maternal dietary intake may regulate reproductive outcomes and the maternal dietary energy has some effects on the lipid metabolism of the offspring [11,17]. We have demonstrated that maternal dietary energy restriction can promote the differentiation of preadipocytes through research with 120% and 80% energy levels of the basal diet during the middle laying period in broiler breeders [15]. While Kurnick et al [18] found that limitation of the dietary intake of pullets from 9 weeks of age to maturity delayed sexual maturity and reduced growth rate. Waaij’s study in broilers also indicated that offspring of mothers that were restricted in their food intake before and around conception may grow to a lower adult weight than chicks of *ad libitum* mothers [19]. Then it is important to confirm the effect of different degrees of restriction on lipid metabolism. So, this study investigated the effect of maternal dietary energy restriction intake with 80%, 70%, and 50% energy level diets of the basal diet on lipid metabolism indexes, such as embryonic deposition of lipid, development of adipocytes, and LPL in embryonic tissues during the medium laying period in AA broiler breeders.

It was reported that maternal dietary energy restriction can influence the hatchability of the hen’s eggs, and most of the reports indicated that more than 85% energy-restricted intake of the hens could keep the quality of the eggs and the hatchability equal to the 100% energy intake group [4,20]. Our results of the 80% and 70% energy-restricted groups might be same with these reports, and the reduction of the hatchability of the 50% energy-restricted group might be due to the increase of mortality rate of embryos.

It has been well-documented that dietary energy has a significant linear effect on the egg weight, increasing energy could significantly increase the egg weight, and a light egg weight was associated with a decreased energy intake [15,21]. These reports were in accordance with the result that the embryo weight at 21 E was significantly decreased with decreasing energy intake. This effect may be because the maternal dietary energy restriction reduces the deposition of nutrients in the egg, while the growth of embryo requires nutrient absorption from the egg. By contrast, the result that the 80% energy group at 13 E was increased compared to the other groups illustrated that the 80% energy restriction might stimulate embryonic development, and this stimulation will fade in the late stage of development. In addition, the relative of relative weight of yolk and relative weight of egg white are also important indexes. The result that energy restriction has no significant effect on the relative weight of eggshell, and yolk relative weight could be decreased the relative weight of egg white could be increased with the decrease of the energy, was in accordance with that maternal dietary energy restriction could have a significant effect on the relative weight of egg yolk and relative weight of egg white [22,23].

It has been estimated that yolk lipids are very important for the growth of chicken embryos because the lipids of the egg are in the yolk to sustain and nourish the developing embryonic chick [24]. Lipids are directly involved in signal transduction as lipid mediators, including phospha-tidylinositols, sphingolipids and eicosanoids [25], and lipids potentially serve as signaling molecules that help coordinate fundamental events during embryo development, implantation, and post-implantation growth [26]. It is known that a lower maternal dietary intake energy could decrease the abdominal fat percentage of the offspring of broider breeders. Adult male offspring of dams fed a high-fat diet during pregnancy and lactation exhibited an increased fat depot weight and liver triglyceride content in the offspring 1 week after weaning as well as in adults [27]. Cholesterol is an essential component of antenatal development, and it is a fundamental mediator of metabolism through the propagation of signaling cascades [28]. Cholesterol is also the precursor of steroid hormones, such as progesterone, and of metabolic mediators, such as oxysterol [29]. In addition, cholesterol is essential to both the activation and propagation of hedgehog signaling; sonic hedgehog (SHH) is responsible for the patterning and development of the central nervous system [30]. An absolute requirement for fetal cholesterol was established when de novo cholesterol synthesis was absent. For example, the rat result demonstrated that the fetus received little or no cholesterol from the mother and that it instead satisfied its need for cholesterol during fetal development through local synthesis [31]. While the transport of maternal cholesterol to the embryo has not been sufficiently studied, it is reported that modifications in cholesterol synthesis by fat type depend on the energy intake level [32]. Most of the cholesterol in the fetus could be accounted for by synthesis in all fetal tissues. It has been confirmed by recent studies in humans, rodents, and cell cultures that circulating maternal cholesterol can affect fetal metabolism and sterol accretion [29]. It was reported that chicks from high cholesterol hens were able to either catabolize more cholesterol or synthesize less cholesterol or both [33], which was in accordance with our result that low daily feed intake groups increased the yolk cholesterol. Furthermore, there is an age effect that can be explained: during embryonic development, they build up their energy reserves. Part of this is used for the hatching process but still a reserve remains at hatch. Our results that the total cholesterol levels on days 19 E and 21 E were abruptly reduced compared to the previously tested days demonstrated that yolk cholesterol is mainly utilized during the final week of incubation of embryo development, and this is in accordance with the report that the chicks emerge from eggs with large deposits of cholesterol in plasma and tissues, and these deposits are quickly depleted during the first week after hatching [34]. The reports that cholesterol metabolism in developing embryos and posthatch chicks is influenced by cholesterol in both maternal and chick diets and that low daily feed intake could increase the yolk cholesterol demonstrated why the total cholesterol restriction groups were higher than the levels found in the control group in our experiment.

It was reported that appropriate reduction of maternal dietary energy made chickens fattier and increased the antioxidant capacity of offspring muscles [35]. Li et al [36] found that lower maternal energy diets of broiler breeders could increase the percentage of abdominal fat and liver fat, fat content of the breast muscle and subcutaneous fat thickness in offspring. However, our results showed that the crude fat levels in the energy restriction groups in breast muscle on days 17 E and 19 E, in thigh muscle on day 21 E and in the liver on day 15 E were significantly decreased, which was in contrast with this result. Additionally, this result might be because the lower energy intake affects the decomposition reaction during rapid embryonic growth, which is in accordance with that maternal with higher intake of fat exhibited an increased fat depot weight and liver triglyceride content in the offspring [27].

It is widely known that most of the lipid in the yolk, especially cholesterol, will be absorbed into embryonic tissues during rapid embryonic growth [37]. The considerable accumulation of cholesteryl esters, mainly cholesteryl oleate, which is present in the liver during embryo development, arose from synthesis in the yolk sac membrane. This suggested a specific role in the transport of yolk lipids into the embryo and accumulation of cholesterol in the liver [38]. In our study, the content of total cholesterol in the liver on days 13 E and 15 E was also significantly decreased by the energy restriction groups, which might demonstrate that the needs of cholesterol in different growth period were different. In addition, breast and thigh muscle tissues are actively involved in cholesterol metabolism. In this study, the 80% energy group significantly increased the content of total cholesterol in the breast muscle on day 13 E, increased the total cholesterol content in the thigh muscle on day 15 E and decreased the total cholesterol content in the thigh muscle on day 19 E. This result suggested that the 80% energy group could increase the cholesterol metabolism.

The growth of adipose tissue is a comprehensive increase in the number of fat cells and fat cell volume, while fat deposition is mainly the process of fat cell volume enlargement instead of an increase in the number of fat cells. The number of adipocytes appears to be correlated with the body size, and the size of the adipocyte is closely related to the fat content of the live bird. Newcombe et al [39] reported that the adipocyte numbers and mean size responded to the dietary treatments. Additionally, Zhong et al [40] reported that reduced abdominal fat in the restricted broilers is attributed to the reduction in the adipocyte volume, which may be due to the decreased lipogenesis. Additionally, lipid droplets are known to play a very important role in lipid storage in adipocytes or other tissue cells. The result that the maternal energy levels affected the size of the lipid droplet, but had no influence on the number of droplets, was in accordance with that maternal feed restriction can regulate embryonic growth, thus affecting the descendants of the deposition of fat and fat cell morphology [41]. This indicated that maternal feed restriction in the broilers might be attributed to increasing of fat size rather than the number of fat cells.

LPL is important in lipid metabolism. LPL is the rate-limiting enzyme for importing triglyceride-derived fatty acids by muscle for utilization and by adipose tissue for storage [42]. Synthesis of LPL is controlled by regulating the level of LPL mRNA, which in turn, is regulated at the initiation of transcription. Zhao et al. reported that the expression of lipogenic enzyme genes in the liver in broiler chickens exhibited scheduling during embryogenesis [43]. This might be attributed to the result that the relative LPL expression in breast muscle in the 80% energy group showed an increasing trend at 19 E while there was little difference at 17 E. Bergo et al [44] suggested that the increased expression of LPL mRNA might increase release of fatty acids and reduce fat synthesis in adipose tissue. This indicated that the increased expression of LPL mRNA was related to the high catabolism of fat. Then the result of relative LPL expression in breast muscle in the 80% energy group in this study indicated that the maternal energy levels might change the relative expression of LPL in breast muscle, which altered the LPL synthesis rates, promoted adipocyte lipolysis, and then decreased adipocyte area in the breast. Additionally, these results are in accordance with the TEM results, which showed decreased area of adipocytesin in the breast, thigh, and liver tissue.

## CONCLUSION

These results indicated that maternal dietary energy restriction during the middle laying period directly affected the lipid metabolism of embryos. Additionally, on the premise of ensuring the laying rate and hatching rate, the 80% energy restriction increased breast muscle weight, thigh muscle weight and the cholesterol metabolism, decreased the levels of crude fat in tissues as well as decreased the long axis and area of adipocytes in breast muscle, thigh muscle and the liver, which suggested that the 80% energy restriction of the hens might promote the lipid catabolism of embryos.

## Figures and Tables

**Table 1:** Composition and nutrient levels of the experimental diets in Arbor Acres broiler breeders

Items	Control group	80% energy group	70% energy group	50% energy group
Ingredients (%)				
Corn	62.04	48.02	37.98	18.00
Soybean meal	26.10	29.20	31.08	34.98
Soybean oil	2.00	-	-	-
Salt	0.17	0.17	0.17	0.17
CaHPO_4_	1.50	1.60	1.65	1.80
Limestone	7.70	7.60	7.60	7.50
DL-Methionine	0.06	0.08	0.09	0.12
Choline chloride	0.10	0.10	0.10	0.10
Premix^1)^	0.33	0.33	0.33	0.33
Rice hull powder	-	12.90	21.00	37.00
Total	100.00	100.00	100.00	100.00
Nutrient levels				
ME/(MJ/kg)	11.70	9.36	8.19	5.85
CP (%)	17.00	17.00	17.00	17.00
Lys (%)	0.80	0.80	0.80	0.80
Met+Cys (%)	0.64	0.64	0.64	0.64
Ca (%)	3.32	3.32	3.32	3.32
AP (%)	0.46	0.46	0.46	0.46
Nutrient levels^2)^				
EE (%)	4.689	2.283	1.958	1.313
CF (%)	2.533	2.491	2.441	2.352
NFE (%)	36.01	34.228	32.722	29.825

ME, metabolizable energy; CP, crude protein; AP, available phosphorus; EE, ether extract; CF, crude fiber; NFE, nitrogen free extract.

1) The premix provided the following per kg of diets: Vitamin A 12,000 IU, Vitamin D 2,400 IU, Vitamin E 30 IU, Vitamin K 1.5 mg, cobalamin 0.012 mg, thiamine 2.0 mg, biotin 0.20 mg, folic acid 1.2 mg, nicotinic acid 35 mg, pantothenic acid 12 mg, pyridoxine 4.5 mg, riboflavin 9 mg, Fe 80 mg, Cu 8 mg, Mn 100 mg, Zn 80 mg, I 1.0 mg, Se 0.30 mg.

2) Nutrient levels are calculated values.

**Table 2:** Feed allocations of broiler breeder hens from 21 to 60 weeks of age

Age (wk)	Feed allocation (g/d bird)
21	110
22	115
23	120
24	125
25	130
26	142
27	155
28–33	166
34	165
35–36	164
37–38	162
39–44	160
45–54	156
55–60	153

**Table 3:** Nucleotide sequences of primers for real time polymerase chain reaction

Gene	Primer (from 5′→3′)	Fragment length (bp)	Genbank no.
*GAPDH*	PF: 5′-GCCATCACAGCCACACAGA-3′	120	NM_204305
	PR: 5′-TTTCCCCACAGCCTTAGCA-3′		
*LPL*	PF: 5′-GACGGTGACAGGAATGTATGAAAG-3′	101	EU477529
	PR: 5′-GAACCAGCCAGTCCACAACA-3′		

*GAPDH*, glyceraldehyde-3-phosphate dehydrogenase; *LPL*, lipoprotein lipase.

**Table 4:** Effects of energy restriction on laying performance of broiler breeders

Item	Control group	80% energy group	70% energy group	50% energy group	SEM	p-value
Laying rate (%)	70.89^a^	63.77^a^	47.43^b^	29.24^c^	2.71	<0.01
Qualified egg rate (%)	90.95^b^	95.12^a^	93.68^a^	95.27^a^	0.43	<0.01
Crack egg rate (%)	3.50^a^	2.87^b^	2.83^b^	2.14^c^	0.12	<0.01
Egg weight (g)	67.61^a^	65.79^b^	65.23^b^	64.25^b^	0.26	<0.01

SEM, standard error of the mean.

a–c Means with different superscripts within each row are significantly different (p<0.05).

**Table 5:** Effects of maternal dietary energy restriction on hatching performance

Items	Control group	80% energy group	70% energy group	50% energy group
Fertilization rate (%)	88.3^b^	94.30^a^	92.84^a^	83.68^c^
Hatching rate (%)	82.04^a^	85.02^a^	83.03^a^	74.00^b^
Healthy chick rate (%)	99.69	99.73	99.75	99.68

a–c Means with different superscripts within each row are significantly different (p<0.05).

**Table 6:** Effects of different energy restriction on crude protein, crude fat and cholesterol of eggs

Item	Control group	80% energy group	70% energy group	50% energy group	SEM	p-value
n	10	10	10	10		
Crude protein content in yolk (%)	31.39	30.61	30.43	30.80	0.13	0.06
Crude fat content in yolk (%)	52.91	52.60	52.48	52.63	0.27	0.96
Total cholesterol content in yolk (mmol/kg)	64.09^a^	64.22^a^	48.14^b^	61.06^a^	1.45	<0.01
Crude protein content in egg white (%)	78.90^a^	78.83^ab^	78.76^ab^	77.87^b^	0.14	0.02
Crude fat content in egg white (%)	2.23^ab^	2.36^a^	2.06^b^	2.17^b^	0.03	<0.01

SEM, standard error of the mean.

a,b Means with different superscripts within each row are significantly different (p<0.05).

**Table 7:** Effects of maternal energy restriction on egg components and egg solids

Item	Control group	80% energy group	70% energy group	50% energy group	SEM	p-value
n	10	10	10	10		
Relative weight of yolk (%)	32.04^a^	29.17^b^	28.06^c^	26.84^d^	0.36	<0.01
Relative weight of egg white (%)	56.31^d^	58.21^c^	59.22^b^	60.47^a^	0.30	<0.01
Relative weight of egg shell (%)	12.06	12.57	12.58	12.81	0.13	0.30
Dry matter content in yolk (%)	54.13	54.03	54.29	54.03	0.13	0.87
Dry matter content in egg white (%)	13.12	13.26	13.31	13.14	0.39	0.61

SEM, standard error of the mean.

a–d Means with different superscripts within each row are significantly different (p<0.05).

**Table 8:** Effects of maternal energy restriction on indexes of growth and development of embryos

Item	Control group	80% energy group	70% energy group	50% energy group	SEM	p-value
Embryo weight/egg weight (%)						
n	10	10	10	10		
13	10.90^b^	13.51^a^	11.41^b^	11.49^b^	0.28	<0.01
15	22.17	21.38	21.47	21.81	0.39	0.90
17	33.62	35.77	34.79	35.33	0.51	0.50
19	47.03	46.65	48.41	49.03	0.75	0.66
21	72.12^a^	69.49^b^	66.34^c^	65.07^c^	0.59	<0.01
Breast muscle weight (g)						
n	10	10	10	10		
13	1.04^b^	1.32^a^	1.17^ab^	1.08^b^	0.03	0.02
15	1.84	1.80	1.80	1.79	0.05	0.99
17	2.44	2.46	2.45	2.46	0.05	1.00
19	3.24	3.52	3.32	3.30	0.07	0.54
21	4.25	4.24	4.28	4.19	0.07	0.98
Thigh muscle weight (g)						
n	10	10	10	10		
13	1.69^b^	2.19^a^	1.65^b^	1.64^b^	0.07	<0.01
15	3.03	2.72	2.75	2.70	0.08	0.50
17	4.52^b^	5.50^a^	5.20^ab^	4.98^ab^	0.11	<0.01
19	7.04	6.83	6.56	6.46	0.12	0.30
21	10.96	10.86	10.45	10.35	0.13	0.27
Liver weight (g)						
n	10	10	10	10		
13	0.30	0.36	0.34	0.35	0.01	0.33
15	0.69	0.72	0.69	0.68	0.01	0.46
17	1.07	1.17	1.02	1.04	0.03	0.38
19	1.32	1.29	1.35	1.37	0.02	0.68
21	2.23	2.24	2.42	2.51	0.05	0.11

SEM, standard error of the mean.

a,b Means with different superscripts within each row are significantly different (p<0.05).

**Table 9:** Maternal energy restriction on contents of crude fat, total cholesterol and gross energy in yolk from E 13 onwards

Item	Control group	80% energy group	70% energy group	50% energy group	SEM	p-value
Crude fat (%)						
n	10	10	10	10		
13	52.12	53.75	52.11	52.74	0.44	0.53
15	51.87	51.27	50.05	50.48	0.52	0.63
17	40.93	41.99	40.76	40.98	0.49	0.83
19	23.60	24.48	22.69	23.55	0.55	0.74
21	18.44	19.03	19.09	18.61	0.52	0.97
Total cholesterol (mg/g)						
n	10	10	10	10		
13	12.01^b^	15.00^a^	14.24^a^	14.53^a^	0.4	0.03
15	11.47	11.90	11.30	11.48	0.21	0.79
17	7.08^b^	8.09^a^	8.24^a^	8.53^a^	0.17	<0.01
19	6.33	7.32	6.40	6.58	0.23	0.43
21	4.79	5.10	5.10	4.56	0.14	0.46
Gross energy (MJ/kg)						
n	10	10	10	10		
13	39.06	39.12	39.75	39.90	0.34	0.77
15	38.82	38.85	39.00	38.76	0.35	1.00
17	37.59	37.48	37.50	37.06	0.38	0.97
19	37.14	36.93	36.92	37.00	0.30	0.99
21	35.45	36.00	35.78	35.28	0.33	0.89

SEM, standard error of the mean.

a,b Means with different superscripts within each row are significantly different (p<0.05).

**Table 10:** Effects of maternal energy restriction on contents of total cholesterol and crude fat in embryo tissues from E 13 onwards

Item	Control group	80% energy group	70% energy group	50% energy group	SEM	p-value
Crude fat in breast (%)						
n	10	10	10	10		
13	1.02	1.02	1.12	1.03	0.02	0.43
15	1.32	1.06	1.14	1.03	0.05	0.19
17	1.80^a^	1.43^b^	1.53^b^	1.49^b^	0.04	0.01
19	2.87^a^	2.48^b^	2.52^b^	2.50^b^	0.06	0.03
21	2.81	2.81	2.79	2.77	0.09	1.00
Cholesterol in breast (%)						
n	10	10	10	10		
13	2.02^b^	2.58^a^	2.08^b^	2.09^b^	0.07	<0.01
15	2.10	2.08	2.05	2.03	0.04	0.92
17	3.43	3.61	3.56	3.50	0.05	0.70
19	3.78	3.34	3.34	3.29	0.11	0.33
21	2.65	2.52	2.58	2.60	0.05	0.85
Crude fat in thigh muscle (%)						
n	10	10	10	10		
13	1.10	0.98	1.13	1.04	0.03	0.45
15	2.08	2.00	1.82	1.79	0.08	0.50
17	2.93	3.01	2.92	2.95	0.06	0.96
19	4.90	4.50	4.49	4.46	0.11	0.48
21	5.23^a^	4.74^b^	4.78^b^	4.70^b^	0.77	0.04
Cholesterol in thigh muscle (%)						
n	10	10	10	10		
13	3.41	3.22	3.19	3.20	0.08	0.75
15	3.57^b^	3.83^a^	3.62^b^	3.59^b^	0.33	0.01
17	5.19^a^	4.51^b^	4.55^b^	4.42^b^	0.08	<0.01
19	4.21^a^	3.23^b^	4.29^a^	4.21^a^	0.10	<0.01
21	2.85	2.57	2.49	2.52	0.06	0.15
Crude fat in liver (%)						
n	10	10	10	10		
13	2.18	2.06	2.02	2.00	0.04	0.48
15	3.67^a^	2.72^b^	2.75^b^	2.70^b^	0.11	<0.01
17	5.32	5.41	5.38	5.42	0.08	0.97
19	10.75	11.01	10.66	10.89	0.20	0.94
21	16.68	15.44	15.38	15.62	0.34	0.51
Cholesterol in liver (%)						
n	10	10	10	10		
13	11.62^a^	10.68^b^	10.59^b^	10.60^b^	0.12	<0.01
15	16.62^a^	15.68^b^	15.70^b^	15.73^b^	0.12	<0.01
17	24.17	24.75	24.22	24.30	0.23	0.82
19	29.26	29.69	29.42	29.57	0.26	0.95
21	32.87	33.02	31.28	32.05	0.58	0.71

SEM, standard error of the mean.

a,b Means with different superscripts within each row are significantly different (p<0.05).

**Table 11:** Effects of maternal energy restriction on long axis, area, quantity of breast muscle of embryo from E 13 onwards

Item	Control group	80% energy group	70% energy group	50% energy group	SEM	p-value
Long axis (μm)						
n	10	10	10	10		
13	0.50^a^	0.45^b^	0.40^b^	0.41^b^	0.01	<0.01
15	0.43^a^	0.40^ab^	0.37^b^	0.37^b^	0.01	0.02
17	0.44^a^	0.32^b^	0.31^b^	0.31^b^	0.04	0.04
19	0.44^a^	0.31^b^	0.30^b^	0.30^b^	0.02	0.01
21	0.45^a^	0.32^b^	0.34^b^	0.32^b^	0.02	<0.01
Area (μm^2^)						
n	10	10	10	10		
13	0.68^a^	0.64^a^	0.48^b^	0.49^b^	0.03	0.01
15	0.56	0.54	0.49	0.49	0.02	0.71
17	0.61^a^	0.42^b^	0.41^b^	0.41^b^	0.03	0.01
19	0.62^a^	0.42^b^	0.40^b^	0.39^b^	0.24	<0.01
21	0.61^a^	0.44^b^	0.43^b^	0.43^b^	0.03	<0.01
Quantity (number/27 μm^2^)						
n	10	10	10	10		
13	13.25	13.83	13.44	13.29	0.70	0.99
15	13.95	14.00	13.88	13.90	0.59	1.00
17	14.62	16.91	15.70	16.01	0.86	0.84
19	16.19	16.23	16.02	16.00	0.46	1.00
21	16.18	16.10	16.00	16.06	0.37	1.00

SEM, standard error of the mean.

a,b Means with different superscripts within each row are significantly different (p<0.05).

**Table 12:** Effects of maternal energy restriction on long axis, area, and quantity of adipocytes in thigh muscle of embryo from E 13 onwards

Item	Control group	80% energy group	70% energy group	50% energy group	SEM	p-value
Long axis (μm)						
n	10	10	10	10		
13	0.36	0.33	0.34	0.33	0.02	0.94
15	0.32	0.35	0.34	0.33	0.02	0.95
17	0.35^a^	0.36^a^	0.29^b^	0.29^b^	0.01	<0.01
19	0.30	0.31	0.30	0.29	0.01	0.91
21	0.72^a^	0.39^b^	0.38^b^	0.38^b^	0.04	<0.01
Area (μm^2^)						
n	10	10	10	10		
13	0.52	0.48	0.50	0.50	0.03	0.96
15	0.43	0.43	0.41	0.40	0.03	0.99
17	0.47^a^	0.48^a^	0.40^b^	0.42^b^	0.01	<0.01
19	0.40	0.42	0.40	0.39	0.01	0.90
21	1.03^a^	0.53^b^	0.54^b^	0.55^b^	0.05	<0.01
Quantity (number/27 μm^2^)						
n	10	10	10	10		
13	19.65	19.39	19.29	19.44	0.96	1.00
15	17.20	16.56	16.88	16.92	1.29	1.00
17	12.71	12.13	12.54	12.62	0.80	1.00
19	7.83	7.00	7.22	7.31	0.56	0.97
21	6.50	6.89	6.60	6.72	0.29	0.97

SEM, standard error of the mean.

a,b Means with different superscripts within each row are significantly different (p<0.05).

**Table 13:** Effects of maternal energy restriction on long axis, area, quantity of adipocytes in liver of embryo from E 13 onwards

Item	Control group	80% energy group	70% energy group	50% energy group	SEM	p-value
Long axis (μm)						
n	10	10	10	10		
13	0.72	0.74	0.71	0.71	0.03	0.99
15	1.43	1.38	1.44	1.37	0.05	0.94
17	2.33	2.30	2.30	2.28	0.11	1.00
19	3.27	2.94	2.92	2.88	0.13	0.73
21	4.54^a^	3.24^b^	3.26^b^	3.29^b^	0.18	0.02
Area (μm^2^)						
n	10	10	10	10		
13	0.97	1.02	0.96	1.00	0.05	0.97
15	2.13	1.99	1.95	1.94	0.07	0.81
17	2.68^a^	2.31^b^	2.30^b^	2.29^b^	0.05	<0.01
19	4.65	4.28	4.27	4.28	0.22	0.92
21	6.55^a^	4.80^b^	4.81^b^	4.78^b^	0.22	<0.01
Quantity (number/27 μm^2^)						
n	10	10	10	10		
13	21.75	21.00	21.37	21.55	0.45	0.95
15	18.00	18.10	18.02	17.55	0.37	0.96
17	16.94	17.29	17.56	17.49	0.39	0.95
19	13.93	13.26	13.24	13.10	0.42	0.91
21	13.14	12.74	12.37	12.55	0.35	0.90

SEM, standard error of the mean.

a,b Means with different superscripts within each row are significantly different (p<0.05).
